# Health technology assessment and priority setting for universal health coverage: a qualitative study of stakeholders’ capacity, needs, policy areas of demand and perspectives in Nigeria

**DOI:** 10.1186/s12992-020-00583-2

**Published:** 2020-07-08

**Authors:** Benjamin S. C. Uzochukwu, Chinyere Okeke, Niki O’Brien, Francis Ruiz, Issiaka Sombie, Samantha Hollingworth

**Affiliations:** 1 Department of Community medicine, College of Medicine, University of Nigeria Enugu Campus Nigeria, Enugu, Nigeria; 2 International Decision Support Initiative (iDSI), Global Health and Development Group, School of Public Health, Imperial College London, Norfolk Place, London, W2 1PG UK; 3 West Africa Health Organisation, Organisation Ouest Africaine de la Santé, 175 avenue Ouezzin Coulibaly, Bobo-Dioulasso 01, 01 BP 153 Burkina Faso; 4 School of Pharmacy, University of Queensland, Wooloongabba, QLD 4102 Australia

**Keywords:** Health technology assessment, Universal health coverage, Stakeholders, Nigeria

## Abstract

**Introduction:**

Health technology assessment (HTA) is an effective tool to support priority setting and generate evidence for decision making especially en route to achieving universal health coverage (UHC). We assessed the capacity needs, policy areas of demand, and perspectives of key stakeholders for evidence-informed decision making in Nigeria where HTA is still new.

**Methods:**

We surveyed 31 participants including decision makers, policy makers, academic researchers, civil society organizations, community-based organizations, development partners, health professional organizations. We revised an existing survey to qualitatively examine the need, policy areas of demand, and perspectives of stakeholders on HTA. We then analyzed responses and explored key themes.

**Results:**

Most respondents were associated with organizations that generated or facilitated health services research. Research institutes highlighted their ability to provide expertise and skills for HTA research but some respondents noted a lack of human capacity for HTA. HTA was considered an important and valuable priority-setting tool with a key role in the design of health benefits packages, clinical guideline development, and service improvement. Public health programs, medicines and vaccines were the three main technology types that would especially benefit from the application of HTA. The perceived availability and accessibility of suitable local data to support HTA varied widely but was mostly considered inadequate and limited. Respondents needed evidence on health system financing, health service provision, burden of disease and noted a need for training support in research methodology, HTA and data management.

**Conclusion:**

The use of HTA by policymakers and communities in Nigeria is very limited mainly due to inadequate and insufficient capacity to produce and use HTA. Developing sustainable and institutionalized HTA systems requires in-country expertise and active participation from a range of stakeholders. Stakeholder participation in identifying HTA topics and conducting relevant research will enhance the use of HTA evidence produced for decision making. Therefore, the identified training needs for HTA and possible research topics should be considered a priority in establishing HTA for evidence-informed policy making for achieving UHC particularly among the most vulnerable people in Nigeria.

## Introduction

Universal health coverage means that all personswho need health services get quality health services without suffering financial hardship [1]. In 2014, The Nigerian government affirmed that UHC is key to ensuring equitable access to high-quality, affordable health care for all Nigerians and Nigeria is a signatory to the UHC agenda and health-related Sustainable Development Goals (SDGs). In response the Federal and States’ Ministries of Health, in collaboration with development partners and other stakeholders developed the second National Strategic Health Development Plan (NSHDPII) 2018–2022. The focus is to ensure that Nigerians have universal access to comprehensive, appropriate, affordable and quality essential health care through revitalization of primary health care (PHC). The plan included high-impact cost effective interventions and mechanisms for quality of data management and governance, as well as a detailed monitoring and evaluation strategy [2].

In addition, the National Health Act of 2014allocated adequate public resources to health for strengthening PHC through the Basic Healthcare Provision Fund. The National Health Insurance Scheme (NHIS) will manage 50% of the fund to ensure access to a minimum package of health services for all Nigerians and the National Primary Healthcare Development Agency will manage 45% of the fund for upgrading and maintaining primary health care facilities, provision of essential medicines, and recruitment and deployment of health workers to primary healthcare facilities. The Federal Ministry of Health will manage the remaining 5% for national health emergencies and responses to epidemics [3]. A new policy of ‘Primary Care Under One Roof’ was also part of the intervention for achieving UHC [4]. And most recently the National Assembly had passed the National Health Insurance Commission Bill to put the country on the path of achieving UHC.

Despite these commitments to UHC, progress has been slow [5]. In Nigeria, there is still a high out-of-pocket expenses for healthcare, a very low budget for health at all levels of government, and poor health insurance penetration and heavy reliance on out-of-pocket payments [4, 6]. The dwindling scarce resources in most low- and middle-income countries (LMIC), including Nigeria, cannot ensure that everyone obtains health services at an affordable price. Therefore, priority-setting processes are necessary to maximise the health system’s ability to work towards UHC due to the unrelenting issue of limited healthcare resources [7] and insufficient health revenue to satisfy increasing and competing demands [8, 9]. They will enable the provision of a comprehensive range of key services that are well aligned with other social goals [10]. Many of the most effective interventions that favour the poor continue to be under-used, while less cost–effective and sometimes wasteful interventions are funded [11].

At present, health care resource allocation in Nigeria is in favour of secondary and tertiary care at the detriment of PHC [12, 13]. Hence, most people bypass PHC facilities and directly seek primary care at secondary and tertiary facilities. Although different methods of priority setting in health care have been used [14], evidence-informed deliberative processes are needed, including cost effectiveness analysis [15]. Nigeria needs an inclusive, standardised healthcare priority-setting process and health technology assessment (HTA) is a useful method for priority setting for more efficient allocation of resources [7] and achieving UHC in a resource constrained environment [16].

HTA is defined as a multidisciplinary process that summarizes information about the properties, effects and/or impacts of health technologies and interventions [17]. It is a policy tool and its main purpose is to generate evidence to inform policy decisions and practice [18]. It is increasingly recognized as a useful policy tool in LMICs, where evidence is needed to guide UHC policies like quality improvement interventions and quality standards [18]. It is also an essential part of a well-functioning and performing health system [19].

There is a growing momentum towards UHC and ‘PHC for all’ in Africa. With limited resources and growing pressures on healthcare systems, sound decision-making and effective prioritisation will be crucial [20]. This is where HTA comes in – the WHA resolution on HTA in 2014 provides an important mandate for all member states [21]. HTA helps identify ineffective practice as well as supporting the careful diffusion of new technologies as health insurance systems develop on the path to UHC. HTA can also help identify cost-effective services that could be delivered at PHC level and support arguments to redirect resources for better prevention and screening activities [22]. In addition, the issue of the transition agenda – countries having to subsidize (or not) products and services previously supported by development assistance funds - implies tough priority setting decisions which ideally should be informed by evidence (e.g. HTA).

At present, health technology assessment and appraisal is fragmented across Africa. Often small teams, based in health ministries and without explicit remit (and inadequate resources), are conducting HTA in an ad hoc way. It is necessary to bring these users and producers of HTA evidence together perhaps through some sort of ‘community of practice’ (e.g. this group in francophone Africa https://www.thecollectivity.org/en/communities/cop-ets). The current lack of HTA in sub-Sahara Africa (SSA) can be attributed to lack of political support for HTA [23], the level of skills to conduct HTA [24], and the availability of data to support HTA [25].

In most sub-Saharan Africa, HTA considerations in decision-making processes are mostly non-existent [7]. Developing sustainable and institutionalized HTA systems requires both technical and non-technical in-country expertise and active participation from a range of stakeholders [26], including (government) decision makers, clinicians, academics, consumers, development partners, and HTA knowledge brokers [27]. However, the biggest challenge is to develop the capacity to use HTA information hence the need to establish technical expertise for HTA to meet health system demands and apply it in all relevant decision-making processes. The use of HTA by policymakers and communities in Nigeria is very limited, hindered by inadequate and insufficient capacity to produce and use HTA and knowledge of the use [28].

Recognising that there is insufficient information on stakeholders’ capacity and needs to produce and use HTA to inform priority setting in health, we aimed to assess the capacity, needs, and perspectives of stakeholders in maternal, neonatal and child health (MNCH) for HTA.

## Methods

### The context of health system in Nigeria

Nigeria is a lower middle-income country with a population of 174 million and a gross domestic product of US$522 billion [29]. It is a federation of 36 states and one federal capital territory with 774 local government areas (LGA). The health care system comprises of a large public sector and private sector provision of health services. The health system mirrors the three tiers of government (e.g. federal, state and local); each has substantial autonomy overlapping roles leads to duplication of effort, wastage, or total neglect [6]. The federal government provides wide-ranging support to state and local governments on health program planning and implementation [2]. The Federal Ministry of Health (FMoH) is responsible for health policy-making, national healthcare priority setting and overall stewardship and leadership for health and provision of tertiary health care [30]. The State Ministries of Health (SMoH) provide health care services through secondary level health facilities, as well as technical assistance to the LGA Health Departments. The LGAs have the responsibility for the Primary health care level of care. There are more than 34,000 health facilities, 66% of which are owned by the three tiers of government (federal, state, and local) [31]. The secondary and tertiary level health facilities are mostly found in urban areas, whereas rural areas are predominantly served by PHC facilities.

### Study design and participants

We surveyed 31 participants in a national workshop organised by the Federal Ministry of Health and supported by West African Health Organisation titled *“Nigeria Research Days for Maternal Neonatal and Child Health (MNCH): implementation of moving MNCH evidence into policy”* 11–13 July 2018 in Abuja, Nigeria. The participants included decision makers who have power to influence policy prioritization, policy makers, academic researchers, civil society organisations, community-based organisations, development partners, and health professional organisations; i.e. those who have an interest in how HTA can improve priority setting in MNCH and potential suppliers of HTA-relevant data. Most of these respondents were all stakeholders in MNCH and were also participants in the meeting. Majority of them participated in the survey.

### Data collection

We revised an existing survey (12 questions), to examine stakeholders’ capacity, need, and perspectives (including supply) of HTA and delivered it to the participants. The survey was anonymous and self-reported; participants were given ample time to complete the questionnaire.

### Data analysis

The data was analysed with a grounded theory approach, where key themes were identified by the authors based on inductive reasoning of participant responses. We further grouped the key themes using the conceptual framework as set out elsewhere [32] where we defined countries’ “priority-setting readiness” from 1)the supply side – the country’s capacity for priority-setting, in terms of institutional capacity, human capacity, evidence or data capacity; 2) the demand side – whether there is an articulated demand from policymakers for active and explicit priority-setting; and 3) the level of need – the potential gains from implementation of explicit and active priority-setting, for example absolute and relative gains in terms of health outcomes and financial protection, taking into account scale and applicable population. Two authors thematically analysed the open-ended questions in three stages using Excel (Microsoft Office): (i) reviewing all the textual data to gain an overall impression; (ii) identifying all comments that appeared noteworthy to the research, extracting these initial themes; and (iii) collating and synthesizing primary themes.

For rating of the policy areas in which the output from a HTA process was needed and types of health technology in which the output from a HTA process was urgently needed we used a forced ranking system where 6 and 7 options, respectively, were ranked in order of their importance as compared with each other. For the level of respondents’ interest in the use of different types of HTA outputs we used a Likert scale of 1–10, where each HTA output was rated 1–10 on level of interest. For the rating of importance of attributes of HTA we used a Likert scale of 1–10, where each policy attribute was rated 1–10 on importance. We then descriptively analysed frequencies, proportions and the mean of rankings using IBM SPSS (Version 24.0).

## Results

### Characteristics of respondents

We recruited 31 respondents but only 25(81%) completed the questionnaire. Participants were from the Federal Ministry of Health, Ministries, Departments and Agencies (MDA, parastatals), Professional Associations, teaching hospitals, health regulatory bodies, research institutions, the academic community, development partners, Non-government organisations, and the regional West Africa Health Organisation (Table 1). The survey took about 40 min but some participants required longer, depending on the level of detail provided in their short answers.

**Table 1:** Respondents’ Organization

Type of Organization	Number	Percent
Federal Ministry of Health	9	25.7
Parastatals/Ministry Department Agencies	7	20.0
Professional Associations	5	14.3
Regulatory bodies	1	2.9
WAHO Researchers	4	11.4
Research Institutions	2	5.7
Development partners	1	2.9
NGOs	5	14.3
CSO	1	2.9

^a^Multiple choice answer

#### Stakeholder capacity assets of HTA

Most respondents were associated with organisations that generated or facilitated health services research. Research institutes highlighted their ability to provide expertise and skills for HTA research but some respondents noted a lack of awareness of HTA and human capacity for HTA. Political support was regarded as essential but could be impeded by politicised decision-making, internal politics in the leadership of the HTA process, cultural barriers in data and information sharing, and a lack of funding for HTA activities.

### Generators of evidence

Participants identified organisations that generate or supply evidence to support health policy decisions in Nigeria at the international, regional and national levels. International multi-lateral organisations include the World Health Organization and West African Health Organisation (WAHO). Non-governmental organisations (NGOs) were identified as generators of evidence. The national organisations included the Federal and State Ministries of Health, as well as the Federal Ministries of Education, Finance, and Water Resources. Health professional organisations were considered generators of evidence and included the Society of Gynaecology and Obstetrics of Nigeria, Association of Public Health Physicians of Nigeria, and the Medical and Dental Council of Nigeria. The academic sector and National Bureau of Statistics were also noted as suppliers of evidence together with hospitals and health facilities, and health training institutions. Those who generated health services research did so in several ways. Those from the academic sector did so through special surveys, grants secured from their universities and, grant-awarding international organizations. The health professional organizations and NGOs also generated research from grant-awarding international organizations. Some also generated research evidence on their own while the the federal and state ministries and WAHO generated research from their own organizations as well as commissioned researches.

### Users of evidence

The key users of HTA information were the ministries of health, other government departments, public health insurance bodies, providers and health professionals, universities and research institutes, donor organisations, and pharmaceutical companies.

### The strengths and weaknesses of the organisations to generate and use HTA evidence

The strengths of the organizations to generate and use HTA evidence included capacity building for health providers, highly trained personnel and human resources, determined organisation and staff, a good understanding HTA needs, a readiness to utilize evidence for policy making, experienced researchers, strong partnerships, availability of policies and guidelines, use of technology for research, and availability of service delivery data. The weaknesses encompassed inadequate number of health care workers; unmotivated health care workers; lack of funds; weak infrastructure and HTA capacity; low uptake of HTA; weak political will for HTA and lack of software to analyse research data.

### Training and research needs

Five main areas of training needs for HTA generators and users were identified: 1) research methods in HTA and data gathering (and economic evaluations); 2) identifying and implementing evidence and using it to inform policy; 3) conducting economic evaluations; 4) developing capacity and building awareness and 5) Data management. Some respondents noted that both HTA generators and health policy-makers and practitioners need training in HTA to facilitate the reliable and efficient interpretation and use of research results, translation into policy, and advocacy and communications.

There is a need for research in: 1) health system financing (financing schemes, medicine pricing, and the design of sustainable essential benefits packages); 2) health service provision taking into account equity, efficiency, quality; 3) burden of disease (antibiotic medicine resistance, non-communicable disease, childhood immunisation); and 4) health policy research.

### Public and wider civil society – their role in priority setting and decision making

Four themes emerged when respondents were asked to consider the role of the public and wider civil society in priority setting and decision making: 1) the extent of public involvement in consultation processes; 2) the role of advocacy; 3) demand for accountability on health and 4) the absence of any public role in priority-setting decisions. Many respondents stressed the importance of consulting the public, but noted that in practice there was no involvement especially of the civil society organizations in priority setting. Some respondents noted that these groups could adopt an advocacy role by holding decision and policy makers accountable for their roles, and creating pressure through media campaigns to highlight health systems problems.

#### Policy areas for which HTA is needed

The main policy areas for which HTA was considered as urgently needed were the production of clinical guidelines or disease management pathways (ranked 4.6/6, Table 2), informing the design of the basic health benefits package (HBP, 4.4), ‘informing design of health service delivery (4.1), registration of health technologies (3.9), coverage or reimbursement of individual health technologies (3.7), and provider payment reform or pay for performance schemes (3.4).

**Table 2:** Rating of the policy areas in which the output from a HTA process is urgently needed in Nigeria (From 1 to 6)

Policy Area	Mean	Standard Deviation
Production of clinical guidelines or disease management pathways	4.6	1.46
Informing design of basic package of health benefits	4.4	1.36
Informing design of health service delivery	4.1	1.45
Registration of health technologies	3.9	1.6
Coverage or reimbursement of individual health technologies	3.7	1.35
Provider payment reform or pay for performance schemes	3.4	1.9

Key considerations in ranking clinical guidelines highest was the need for uniformity and standardising protocols, management of disease, and saving costs. This was noted by one of the respondents: *“establishing protocols and guidelines and making them available are likely to save costs of treatment and improve the outcome of a disease”.* For HBP design, it was identifying services and technologies that should be covered but in a way that is financially sustainable’ *“in the face of inadequate resources the basic package should be affordable and respond to the basic health needs of the populace”*, one participant explained. Some respondents noted that the design of health service delivery will enable an environment for the provision of effective and efficient services.

### Technology types that will benefit from HTA approaches

Public health programs or initiatives were ranked as the most important technology type that will benefit from HTA approaches (mean 5.23, Table 3) followed by medicines (4.83), vaccines (4.73), other (e.g. surgical) interventions (4.63), service delivery initiatives or incentives (4.44), medical devices and diagnostics (4.42) and screening or referral programs (4.33).

**Table 3:** Types of health technology in which the output from a HTA process is urgently needed in Nigeria. (Ranking 1–7)

Types of HT	Mean	Standard Deviation
Public health programs or initiatives	5.23	1.89
Medicines	4.83	1.95
Vaccines	4.73	1.65
Other intervention (e.g. surgical procedures)	4.63	2
Service delivery initiatives or incentives	4.44	2.04
Medical devices / diagnostics	4.42	1.9
Screening / referral programs	4.33	1.72

Respondents prioritised public health programmes because they reach large numbers of people and can focus on prevention, reducing the burden of disease and therefore health care expenditure. As noted by a participant, *“It is necessary to put in place disease prevention strategies in public health programmes because of the high disease burden in Nigeria”.* Medicines were considered important for functioning health facilities (often an inadequate supply), patient outcomes, the relatively large budget impact, and use for many high-burden diseases. However, one respondent highlighted *“there is general lack or inadequate supply of medicines and medical supplies in clinics and hospitals.”*

Improving the availability and management of vaccines would help reduce the burden of communicable diseases by building herd immunity. Improving service delivery will lead to better diagnosis, treatment and control of the main disease areas. Medical devices were regarded as important especially given recent advances in healthcare. Screening programs help to reduce the disease burden and bridge primary and specialist health care for large parts of the population, particularly in rural and hard to reach areas.

#### Perspectives on HTA in Nigeria

##### Level of stakeholders’ interest in the use of different types of HTA outputs

When asked to rate the level of their interest in the use of different types of HTA output on a scale of (0–10), respondents indicated the most important were safety issues (8.8); economic issues (8.61); efficacy of technology (8.39); information on technology effectiveness (8.37) and social/ethical concerns such as equity and solidarity (7.5, Table 4). Safety concerns were explicitly linked to the availability and use of generic medicines where quality could not be guaranteed, as well as ensuring safety of patients and healthcare providers. One respondent noted that in the Nigerian context *“security issues can effect stability especially in hard to reach communities and insurgent areas”,* suggesting that safety outside of medical facility is also important consideration in providing care in hard to reach communities and not only safety of medicines. Economics issues were linked to affordability, sustainability, and cost-effectiveness of technologies and limited availability of health resources. Efficacy concerns encompassed quality of care and assuring efficacy before implementation of the programme, whereas social and ethical concerns were focussed on the dignity and rights of users and the need for the vulnerable to have access to health care.

**Table 4:** Level of respondents’ interest in the use of different types of HTA outputs (from 1 to 10)

Use of HTA Output	Mean level of interest	Standard Deviation
Safety	8.8	2.09
Economics (e.g. costs, value for money, budget impact)	8.61	1.64
Efficacy	8.39	2.06
Effectiveness (e.g. from real world evidence)	8.37	2.08
Social/ethical concerns (e.g. equity, solidarity)	7.75	2.14

##### Scope for HTA use in Nigeria (importance of HTA attributes)

In relation to the importance of particular attributes of HTA, respondents’ highlighted (mean rating out of 10) improving the quality of health care (8.7), allocative efficiency (8.4), equity (8.3), budget control (8.0), and transparency in decision making (7.9, Table 5).For improving quality of health care, respondents felt it was important because government was not making health a priority and there was no research. For allocative efficiency, they felt it was important to be able to make use of resources, important for allocating resources based on need and achieving UHC; distribution of services evenly, and bridging the gap in health for all. The main reasons given to promote transparency in decision making were the importance for availability, equity, building trust, reducing corruption, ensuring all projects are funded, social inclusiveness, and provision of better quality service delivery. Budget control was regarded as important for planning, project management, improving health outcomes, effectiveness, and ensuring accountability. The reasons for ranking equity as important included accountability, following protocol for funds and decision making, lack of resources in certain areas and organizations and for disadvantaged groups.

**Table 5:** Rating of importance of attributes of HTA (From 1 to 10)

Policy Attributes	Mean	Standard Deviation
Improving quality of health care	8.7	2.38
Allocative efficiency	8.4	2.51
Equity	8.3	2.29
Budget control	8.0	2.52
Transparency in decision making	7.9	2.66

##### Availability of data for HTA

The reported availability of local data to inform country-specific decisions varied across the six domains of data (Table 6). The better availability was for data on disease profiles (e.g. burden of disease, prevalence, incidence) (52%), followed by medicine prices in public and private providers (35%), while the least available was cost of health services (23%). Data were available with limitations across the six domains of data. The main reasons given were that not all diseases are covered or prioritised in data collection, data collection plus monitoring and evaluation does not have adequate funding, available data are not comprehensive or local, and some partners have data they do not share.

**Table 6:** Availability of six sources of data listed

Sources of Data	Available N (%)	Available with limitations N (%)	Not available N (%)	Total N (%)
**Disease Profile**	13 (52.0)	6 (24.0)	6 (24.0)	25 (100)
**Medicines prices** (public or private providers)	8 (34.8)	7 (30.4)	8 (34.8)	23 (100)
**Medicines use** (e.g. the use of specific medicines in a state, or nationally)	7 (29.2)	9 (37.5)	8 (33.33)	24 (100)
**Activity of hospitals** (e.g. how many times is a particular inpatient or outpatient	7 (30.4)	11 (47.8)	5 (21.8)	23 (100)
**e-Health outcomes** (e.g. what is the average 30 day mortality following admission to a hospital for acute myocardial infarction [heart attack] at an individual hospital.	7 (30.4)	14 (60.7)	2 (8.7)	23 (100)
**Cost of health services** (e.g. costs of treating stroke)	5 (22.7)	13 (59.1)	4 (18.2)	22 (100)

## Discussion

This survey represents the first attempt to map HTA capacity, needs and perspectives in Nigeria. The results of the study suggest several important areas that require attention in order to strengthen the capacity of key stakeholders to undertake and interpret HTA. All respondents were associated with organisations that generated or facilitated health services data and research. Research institutes highlighted their ability to provide expertise and skills for HTA research, but some respondents noted a lack of awareness of HTA and human capacity for conducting and interpreting HTA. This calls for interventions to improve capacity in HTA as deficiencies in knowledge and skills in HTA exist.

The organisations which either generate or supply evidence mostly included government agencies, universities and affiliated research institutes, donor organisations and development partners.

Both federal and state governments were identified as the dominant users of HTA. This is not surprising given the three tiers of government play a key role in healthcare delivery in Nigeria. State governments are free to make their own health policies though they must be in line with the Federal government’s policy, meaning that HTA can be used for decision making both at the Federal and State level. Our finding is similar to the reported use of HTA globally, wherein ministries of health were identified as the main initiators of HTA [33]. In Nigeria the FMOH should be a major partner in HTA with other stakeholders, including the research institutes, universities, civil society organizations and development partners. It is important that the local institutional partner(s) are capable of convening other stakeholder’s in-country, as HTA process requires a multidisciplinary team. Based on our experience, many academic institutions in Nigeria do not interact with governmental decision makers, which limit their ability to contribute to policy and HTA capacity building. However, this can be improved on with awareness creation and inclusion of HTA in the curriculum of academic institutions. This will both make the graduates knowledgeable and fortify them with the skills to contribute to HTA capacity building in any institution they gain employment.

The users of HTA are policy makers, ministries of health; regulatory and procurement agencies, insurance bodies and the universities, while the suppliers of evidence included academia, research institutes and the ministry of health. These groups should also be targeted for training in the understanding and use of HTA evidence for policy making.

Our results show the potential policy areas where HTA can be applied and were categorized in order of perceived importance: production of clinical guidelines or disease management pathways, informing the design of the basic health benefits package, informing design of health service delivery, Registration of health technologies, coverage or reimbursement of individual health technologies and provider payment reform or pay for performance schemes. A key consideration in HBP design was identifying services and technologies that should be covered but in a way that is financially sustainable. In settings with inadequate resources, a basic health package should be affordable and respond to the basic health needs of the population. Part of the planning must ensure that people are aware of the services. Knowing these priority areas will be helpful in devising a relevant HTA capacity strengthening program. The result is similar to the use of HTA in India [34].

It is not surprising that public health programs, medicines and vaccines were the most identified as being critical areas for HTA. This is likely due to the high costs associated with these technologies and the ability to address major disease burdens by developing these areas. However, surprisingly public health programs were prioritized over medicines and vaccines that are usually the exclusive domain of HTA. Decision makers are usually interested in two different financial forces (less budget for more demand and more supply at a higher price) and as a result they tend to channel each request for new investment through an evaluation process, assessing the effectiveness of the new programmes or products in real-life situations and whether the money spent is good value for health and for the healthcare programme as opposed to efficacy from randomised clinical trials; and whether it is worth buying the new asset given the limitations that exist [35]. The WHO road map for access to medicines, vaccines and other health products, 2019–2023, aligns with the outputs that have been identified within the WHO framework including provision of authoritative guidance and standards on the quality, safety and efficacy of health products, access to essential medicines, vaccines, diagnostics and devices for PHC [36].

Improving the quality of health care, allocative efficiency and equity issues were the most identified attributes for HTA use in policy making and this is line with the core principles of UHC. It is interesting and of significance that the quality of health care was considered the most important component of HTA. In most HTAs and economic analyses, allocative efficiency have been considered most important in technology adoption. Yet it has been noted that allocative efficiency may not be the only, or the most important, issue to be considered in technology adoption [37]. It is important to note that HTA emphasizes that the approach seeks to be comprehensive and multidisciplinary. A good HTA therefore will always include a solid review of the clinical efficacy of a technology, and would attempt to understand safety issues from the evidence available. The economics side often employs decision theoretic / operations research type methods to synthesize the available clinical and wider epidemiology evidence, and incorporate resource use/costs/preferences. So HTA approaches allow us to better understand the full ‘value’ that a technology brings, providing a framework for both quantitative and qualitative analysis, with the latter emphasizing the importance of deliberation as a means to way up the clinical and economic evidence vis-à-vis local values and the extent of uncertainty, notions around ‘innovativeness’. Thus, HTA combined with deliberative decision making processes also includes safety, efficacy, effectiveness, and they are all part of the ‘value for money’ argument influencing resource allocation which HTA brings together in a coherent and systematic framework tailored to country needs.

It is also important to assess other external factors that could be impacted by the use of such technology or policy like equity and fairness, budgetary control and transparency in decision making. Equity considerations as mentioned by the respondents is also important as integrating equity consideration in HTA can help decision-makers and policy-makers to better understand the distributional impact of health interventions. Equity consideration in economic evaluations have been conducted on some health technology intervention programmes such as vaccines [38]. These prioritized attributes for HTA use in policy making by the respondents therefore underscores the fact that HTA attributes should be context specific. Further research should therefore focus on more informed contextualized categories for scoring HTA attributes.

The use of HTA to address safety issues, such as low quality medicines and value for money concerns, was seen as important, perhaps reflecting problems in Nigeria related to service quality, safety of patients and healthcare providers and efficiency and use of cost effective technology interventions. The efficacy concerns were linked to quality of care and assuring efficacy before implementation of the programme. Social/ethical concerns were linked to the importance of dignity and rights of users and the need for the vulnerable to have access to health care. The explicit use of evidence by incorporating safety, efficacy, cost effectiveness, budget impact, and social and ethical considerations were also noted in India [39] and Thailand [40]. Dang et al. also point to the role of HTA in bringing efficiencies to the health system in India [41].

The perceived availability and accessibility of suitable local data to support HTA varied widely but in many instances was considered inadequate and limited. There are often data on the burden of disease but its application may be limited due to incomplete or unreliable documentation [42]. The availability of information on burden of diseases and data pertaining to particular population subgroups for which a technology may be applicable is important for HTA. Data on medicines and medicine prices is also important, as availability of medicines has been noted to be a major determinant of use of health facilities in Nigeria [43]. Without these data, the evidence base for HTA will be limited. Technical capacity for HTA in LMICs also relies on data availability and management more widely [44, 45].

The respondents identified a range of topics where further research is needed. In prioritization of health technology assessment topics and commissioning of HTA projects, the research topics derived from key stakeholders should be given preferences instead of using globally derived topics that are not context-specific. It has been noted that using information routinely available in the literature and from the vignettes to select HTA topics could not be used to estimate the absolute value of HTA with any certainty in the selection and prioritization of HTA topics [46]. Most HTA programs have criteria for topic selection, although these criteria have been known not to be always explicit [47]. Therefore, qualitative methods of gathering this information, like surveying stakeholder perspectives, should be considered.

Similarly, the areas of greatest training need for HTA generators and users identified by the participants were broad. Some respondents noted that both HTA generators and health policy-makers and practitioners need training in HTA to facilitate the reliable and efficient interpretation and use of research results, translation into policy, and advocacy and communications. Building technical capacity for HTA can take place in-country, in institutions that have the capacity to do so. Examples of the institutions include the Health Economics and Health Policy units of the Institute of Public Health of the University of Nigeria Enugu campus. This can also occur at both the federal and state level since health policies are decided and implemented at the state level as well as federal levels. However, literature on HTA capacity building in resource-poor countries suggests that political will, involvement of stakeholders, technical and financial support from international partners are crucial in developing training programmes [48–50].

## Limitation of the study

The main limitations of this study include that the survey participants were mainly stakeholders in MNCH issues; their expertise in this area may have biased the responses. The representativeness of the responses from participants may have reflected individual views also rather than the position of the participants’ institutions. We also did not also collect information from patients and other health care workers who are also users of healthcare system as they were not part of our study population. Information from them may have given a more comprehensive information towards policy making. This is a gap for future studies.

## Conclusions

These findings illuminate the current situation, the opportunities (including potential HTA topics), and challenges in using HTA in Nigeria. Introducing evidence-informed priority setting in Nigeria and other LMICs will require support from several stakeholders who produce and use HTA evidence. Capacity development for HTA should be shaped by the country’s policy demands. Stakeholder participation in identifying HTA topics and conducting research will enhance the use of HTA evidence for decision making. There is demand for use of evidence in specific policy areas so, long-term capacity development to encourage evidence-informed priority setting will be worth the effort.

## Data Availability

The datasets used and/or analysed during the current study are available from the corresponding author on reasonable request.

## Abbreviations

FMoH: Federal Ministry of Health
HTA: Health Technology Assessment
LGA: Local Government Area
LMIC: Low and middle income countries
MDA: Ministries Development Agencies
MNCH: Maternal, Neonatal and Child Health
NGO: Non-governmental Organization
NSHDP11: National Strategic Health Development Plan 11
OOP: Out-of-poverty
PHC: Primary Health Care
SMoH: State Ministry of Health
UHC: Universal Health Coverage
WAHO: West African Health Organization
WHO: World Health Organization
